# Therapy and Antifungal Susceptibility Profile of *Microsporum canis*

**DOI:** 10.3390/jof4030107

**Published:** 2018-09-05

**Authors:** Chioma I. Aneke, Domenico Otranto, Claudia Cafarchia

**Affiliations:** 1 Dipartimento di Medicina Veterinaria, Università degli Studi “Aldo Moro”, 70010 Valenzano, Bari, Italy; chioma.aneke@unn.edu.ng (C.I.A.); domenico.otranto@uniba.it (D.O.); 2 Department of Veterinary Pathology and Microbiology, University of Nigeria, Nsukka 410001, Nigeria

**Keywords:** *Microsporum canis*, antifungal susceptibility testing, broth microdilution, E-test and Disk diffusion, antifungal resistance

## Abstract

*Microsporum canis* is a worldwide diffused zoophilic dermatophyte which causes clinical conditions often characterised by multifocal alopecia, scaling, and circular lesions in many animal species, including humans. A large variety of oral and topical antifungal protocols is available for treating *M. canis* infection. However, the efficacy of these drugs and treatment protocols is variable, with treatment failure up to 40% of patients possibly due to resistance phenomena. The lack of standardised reference methods for evaluating the antifungal susceptibility of *M. canis* represents a major hindrance in assessing microbiological resistance in unresponsive clinical cases. Therefore, data about conventional therapy against *M. canis* and the protocols employed to test the antifungal activity of the most commonly employed drugs (i.e., azoles, polyenes, allylamines, and griseofulvin) have been summarised herein. This article focuses on technical parameters used for antifungal susceptibility tests, their effects on the minimum inhibitory concentration value, as well as their clinical implications.

## 1. Introduction

*Microsporum canis* is a zoophilic dermatophyte, the causative agent of human and animal dermatophytosis worldwide [1,2]. In animals, *M. canis* infection has been associated with multifocal alopecia, scaling, and circular lesions [3] and with localised forms in humans, such as for tinea capitis, tinea corporis, tinea pedis, and onychomycosis [1]. The distribution of this fungus might vary considerably, depending on the geographical area and other epidemiological factors (i.e., age, sex, and season) [4,5,6,7]. For example, in human patients older than 16 years, females are more frequently infected than males [8]. In dogs and cats, male and young individuals develop more frequently clinical lesions also according to their breed (i.e., Yorkshire terriers, Jack Russell Terrier, and Pekingese) [4,9]. *M. canis* transmission occurs through direct contact with sick or subclinically infected animals, mainly cats, or with arthrospores, that remain viable in the environment for up to 18 months [10]. Human-to-human infection has been frequently recorded and asymptomatic animals are considered to be spreaders of the disease in about 50% of the infected humans [11]. The clinical manifestations of *M*. *canis* infection in animals are similar to those caused by other dermatophytes or other skin diseases, thus requiring a specific diagnosis for their prevention, treatment, and control [4,5,6,7,12]. Due to the highly contagious nature of *M. canis* infections, treatment protocols are mandatory to prevent potential transmission to other receptive hosts [2]. A variety of oral and topical antifungal agents is available and drugs such as griseofulvin (Gri), terbinafine (TER), itraconazole (IT), and fluconazole (FLZ) are used to cure severe infections in humans and animals [2,13,14]. However, *M*. *canis* infections characterised by recurrence and treatment failure has been recorded in 25–40% of treated patients, potentially due to lack of patient compliance, lack of drug penetration into tissue, variable medication bioavailability, and resistance phenomena [15]. In vitro analysis of the antifungal agents allows comparing different antimycotics, which in turn may assist clinicians in exploring resistance phenomena and choosing an effective therapy for their patients. However, a reference method for testing the antifungal susceptibility of *M. canis* has not been standardised resulting in variable and not comparable susceptibility profiles to antifungal drugs. Data about conventional therapy against *M. canis* and protocols employed to test the antifungal activity of azoles, polyenes, allylamines, and GRI have been herein reviewed and clinical implications discussed.

## 2. Conventional Therapy for Animals and Humans

The choice of a proper treatment is determined by the site and extent of the infection, as well as by the efficacy, safety profile, and pharmacokinetics of the available drugs [16]. A vast range of antifungal classes, such as the first oral imidazole (e.g., ketoconazole-KTZ) and GRI have been used in human and veterinary medicine to treat dematophytoses [17]. Later on, other azoles (i.e., FLZ, ITZ, efinaconazole, and luliconazole), allylamines (i.e., TER, butanafine, and naftifine) and amorolfine, and ciclopiroxolamine were employed [18].

### 2.1. Conventional Therapy in Humans

In humans, topical antifungals are recommended for the treatment of tinea capitis though once monotherapy is discontinued relapses may occur [19,20,21]. For more extensive diseases, successful treatment requires concurrent use of both topical and systemic oral antifungal drugs. Among various options, topical TER for 4 weeks and both TER (250–500 mg/day for 2–6 weeks) and ITZ (100–200 mg/day for 2–4 weeks) are effective to control human infection as tinea corporis/cruris/pedis [22]. However, as for tinea capitis affecting prepubescent children, topical treatments are ineffective since they do not reach the inside of the hair shaft where *M. canis* resides [13]. The results of seven works concluded that TER was more effective for tinea capitis primarily caused by *Trichophyton* species, whereas GRI for that caused by *Microsporum* species [23].

### 2.2. Conventional Therapy in Animals

While both topical and systemic therapies are useful to control human infection, the decontamination of the exposed environments are also required for controlling the infection in animals [2,24]. In animals, topical therapies (i.e., weekly application of lime sulphur, enilconazole, or a miconazole/chlorhexidine shampoo) are currently recommended (see Table 1) [2,25,26,27,28,29,30,31].

Miconazole shampoos are most effective when combined with chlorhexidine (see Table 1) [2,24]. Clotrimazole, miconazole, and enilconazole are also recommended for topical treatment of *M. canis* infections in animals, but not as sole therapy [2]. Systemic antifungal therapy (e.g., ITZ, KTZ, GRI, and TER) targeting the active site of fungal infection (see Table 1) is usually required when extensive lesions are present or when asymptomatic animals are involved [2]. GRI with twice-weekly chlorhexidine/miconazole shampoo was more effective than GRI alone to treat these infections in cats (see Table 1) [27]. ITZ is more effective than GRI in treating these infections because of its quick healing time (i.e., 56 days vs. 70 days) (see Table 1). The high efficacy of TER has been demonstrated in different studies mainly for cats with different dosage and time of resolution (see Table 1). However, the recent isolation of a TER-resistant *M. canis* strain from a feline dermatophytosis in China [32], might indicate the low efficacy of this drug for *M. canis* as previously demonstrated for *T. rubrum* and *T. tonsurans* [33,34].

## 3. Antifungal Susceptibility Profile 

A variety of laboratory methods can be used to evaluate or screen the in vitro antimicrobial activity of an extract or a pure compound of which broth microdilution methods is the gold standard or reference technique to perform antifungal susceptibility, proposed by the European Committee on Antibiotic Susceptibility Testing (EUCAST) and the Clinical and Laboratory Standards Institute (CLSI) [35]. Both institutions (i.e., EUCAST and CLSI) have developed breakpoints for some drugs against *Candida* spp. and *Aspergillus* spp. that are currently used to categorize fungal isolates into (i) susceptible (the drug is an appropriate treatment), (ii) resistant (the drug is not recommended as a treatment), and (iii) intermediate (the drug may be an appropriate treatment, depending on certain conditions) [36]. However even if EUCAST approach has increasingly been used worldwide to test the antifungal susceptibility of fungi, the studies using this approach on dematophytes are mainly focused on *Trichophyton* spp. and rarely on *M. canis*. For this reason, the EUCAST procedures were not the object of this review. Contrarily, there are several reports where the antifungal susceptibility testing of dermatophytes had been evaluated by using either agar or broth macrodilution and microdilution tests (see Table 2, Table 3 and Table 4).

In these reports, the methods employed to evaluate the antifungal susceptibility of *M. canis* are generally an extension of M27-A, M38-A, and M38-A2 guidelines and a modified procedure with Sabouraud glucose broth as medium (see Table 2, Table 3 and Table 4). Results obtained by these studies have shown great variability of MIC values, probably due to the lack of standardization of different parameters that can affect the MIC determination.

### 3.1. Broth Microdilution Procedures

With reference to dermatophytes, a microbroth dilution method for testing antifungal susceptibility profile has been adopted as an amendment to the CLSI M38-A since 2004 [37] and successively an intra- and inter-laboratory multicentre study was conducted to establish that *T*. *mentagrophytes*, MRL1957, and *T*. *rubrum*, MRL666 might be used as quality control reference strains for the dermatophyte susceptibility standard [55]. Furthermore, a large number of studies were published on antifungal susceptibility of dermatophytes by using microdilution procedures but the technical parameters (inoculum type and size, temperature, and duration of incubation) greatly varied among them (see Table 2) resulting in incomparable data. It is well known that MIC can be greatly affected by changes in inoculum size, time and temperature of incubation, media, and endpoint definition [35]. Additionally for dermatophyte, it is also important to select the most appropriate medium that supports conidial growth or inoculum preparation. Jessup and Coll [56] were the first to identify media used to obtain large amount of conidia. Three types of agar media (i.e., potato dextrose agar, Mycosel with 1% yeast extract, and Heinz oatmeal cereal) were compared for their abilities to induce conidiation. The results show that the majority of dermatophytes (i.e., *M. canis*, *T. mentagrophytes*, *T. tonsurans*, and *E. floccosum*) are capable of producing conidia in different media as well as the induction of conidiation, but not *T. rubrum* which is medium dependent [56]. Mycosel with 1% yeast extract and Heinz oatmeal cereal were proposed as media useful to produce conidia for dermatophytes [56]. However, these suggestions were never followed and the medium most frequently used to produce conidia was Potato Dextrose Agar (PDA) mainly when *M. canis* was tested (see Table 2). Temperature and incubation time for conidia production in PDA are also parameters, which largely varied (see Table 2). There is therefore the need to ascertain how these parameters might influence the susceptibility profile of dermatophytes as this has never been demonstrated before. However, an incubation time of 4 days has been productive for the antifungal profile test of filamentous fungi (CLSI), but the low growth rate of dermatophyte might result in an increase in the time of incubation (see Table 2). Previous results showed that 7–14 days at 28–30 °C is necessary for conidiation (see Table 2). After that time, cultures of dermatophytes are usually formed by hyphae, microconidia, and macroconidia [37]. The CLSI guidelines recommend separation of the fungal structures (hyphae and conidia) through sedimentation for 15–20 min and use of the upper part of the suspension for susceptibility testing [35]. However, this sedimentation is not totally efficient in separating the hyphae from the inocula suspension when dermatophytes are tested and a filtration procedure is usually required [57]. It has been shown that the MIC values of the different drugs varied with the structures tested (i.e., hyphae or conidia). For example, highest MIC values were seen when testing drugs with nonfiltered inocula (hyphae + conidia) because of the high thickness of the cell walls [48,57]. Nevertheless, based on published studies, the MIC ranges for FLZ, KTZ, or TER was higher by using conidia than hyphae + conidia (see Table 2) suggesting that the roles of inoculum in influencing the MIC values of *M. canis* need to be further investigated. Interestingly, it has been shown that arthroconidia of *T. rubrum* and *T. mentagrophytes* appear to be more resistant to the drugs than microconidia and may be one of the causes of therapeutic failure, mainly in patients whose lesions contain abundant arthrospores [58]. The remaining parameters (i.e., inoculum size, time and temperature of incubation, media, and endpoint determination) useful to evaluate the susceptibility profile of *M. canis* have been assessed by two multicentre studies demonstrating a high level of intra and interlaboratory agreement for the determination of MICs conducted using RPMI medium [37,59]. However, inoculum size, temperature, and incubation time greatly varied among the above two studies and consequently among successive published papers (see Table 2). Particularly, the inoculum size most commonly employed to evaluate the antifungal susceptibility of *M. canis* ranged from 10^3^ to 10^4^ CFU/mL, temperatures ranged from 27 °C to 35 °C and incubation days from 4 to 14 days (see Table 2). It has been shown that inoculum size of *Trichophyton* spp. might affect the MIC values of some drugs (i.e., ITZ, clotrimazole, FLZ, and GRI), and usually the growth for *Microsporum* spp. ranged from 6 to 10 days at 30 °C [16], but the temperature used highest inoculum size showed the best reproducibility of data among interlaboratory studies [37,59]. For the temperature and incubation time, it is well known that the optimal in previous reports greatly varied ranging from 25 to 37 °C for 4 to 14 days. Based on interlaboratory data, the highest reproducibility of data was observed by incubation for 7 days at 28 °C [59] or 4 days at 35 °C [37]. Thus, the MICs obtained by this methods need to be correlated with clinical outcomes to demonstrate the true value of these data. The last parameter that needs to be clearly ascertained for defining the antifungal susceptibility profile of *M. canis*, is the value to define azole MICs. Specifically, the CLSI for filamentous fungi document recommends 50% inhibition to define azole MICs. However, most stringent MIC determination criterion (MIC-0 = 100% inhibition or MIC-80 = 80% inhibition in respect to the control) has shown the best reproducibility of data among intra and interlaboratories results for defining the antifungal susceptibility of *M. canis* strains [37,59]. Finally, despite the difficulty in comparing MIC results of tested drugs due to variability in the different methods and conditions under which they were evaluated, FLZ seems to be the drug with the lowest activity while TER and ITZ with the highest activity regardless of the method employed. 

### 3.2. E-Test Procedure

The E-test is a simple, agar-based, quantitative minimal inhibitory concentration (MIC) method that is satisfactorily used to test fungi, mainly *Candida* spp. and *Cryptococcus* spp. [35]. This assay has been employed to evaluate the antifungal profile of dermatophytes in different studies, but no guidelines have been published till date for these fungi. However, with exception of one study [47], the parameters employed for testing *M. canis* with E-test procedures (see Table 3) are concordant among the studies: (i) inoculum size of 10^5^–10^6^ CFU/mL; (ii) RPMI agar as medium; and (iii) 3 days at 27 to 28 °C as time and incubation temperature were concordantly used [41,48,49]. In addition, a study in which the susceptibility profile of *M. canis* was evaluated by using E-test through an interlaboratory agreement has confirmed the usefulness of these parameters [48] with high level of agreement for FLZ (i.e., 100%) and moderate agreement for KTZ and ITZ (60%) [48]. The low interlaboratory agreement for KTZ and ITZ were more likely due to the difficulty in reading the MIC end points with E-test as previously reported for other fungi [48]. Furthermore, it was common to find two growth zones of different density around the strip, or the presence of microcolonies inside the inhibition zone when KTZ was used [48]. However, when a very clear ellipse was seen in the plate, the results are usually highly reproducible [60]. By using the E-test with these procedures, FLZ seems to be the drug with the lowest activity and ITZ the most active drug against *M. canis*. The lack of availability data on E-test procedures for POS and VOR as well the lack of E-test strip for TER does not allow the evaluation of effectiveness of these drugs against *M. canis* by using this procedure.

### 3.3. Disk Diffusion Procedures

The disk diffusion method (DD) is an agar-based method characterised by the ease of use, reproducibility, accuracy, and low cost for the detection of antifungal susceptibility profile [61,62]. Guideline for testing filamentous fungi by DD method has been proposed by CLSI M51-A [63], but no agar-based susceptibility testing method has been validated for the dermatophytes. Previous reports are mainly related to *Trichophyton* spp. [54] and a few to *M. canis* (see Table 4). Different parameters were used to perform this test, thus making the comparison of the obtained results very difficult (see Table 4). Particularly, the time of incubation prior to preparation of inoculum, ranged from 4 to 28 days, with an inoculum size of strains from 10^4^ to 10^6^ CFU/mL and different media (RPMI and Mueller–Hinton (MH) agar) are the most frequently employed. The incubation time and temperature for reading the results also varied ranging from 5 to 14 days and from 25 to 30 °C. Although all the parameters above could have a role in influencing the MIC values, to date there is scant report that has addressed parameters that could be considered important for *M. canis* susceptibility testing with the DD assay. Specifically, Fernandez-Torres and Coll [64] were the first to evaluate the best media for DD assay. Three media (i.e., RPMI 1640 medium with L-glutamine and without bicarbonate; antibiotic medium 3; high-resolution medium) were compared and the results were dependent on the drug/species combinations tested. For example, the influence of the culture medium was observed only for *M. canis* and *T.mentagrophytes* when ITZ was tested [64]. However, when the parameters (i.e., pre-incubation time and temperature, inoculum size, media, incubation time, and temperature) employed for DD assay by Fernandez-Torres and Coll [64] were followed by other authors [53], the same MIC range were obtained, thus showing the reproducibility of this method (see Table 4). The role of inoculum size (10^4^–10^6^ CFU/mL), media (i.e., MH agar with methylene blue and RPMI), and reading time (from 3 to 7 days) in influencing DD assay were also studied and optimised [52]. In particular, a pre-incubation time of 14 days at 28 °C on PDA, 1 × 10^6^ CFU/mL as an inoculum size, and MH agar incubated at 30 °C for 7 days for reading the results should be the optimal condition for DD [52]. Despite the variation of the susceptibility profile of *M. canis* according to the parameters employed to perform DD assay, it is most likely that VOR and TER are the drugs with the highest inhibition zone regardless of the parameters employed, (see Table 3) thus, being promising as potential antifungal drugs in the treatment of recalcitrant superficial mycoses due to *M. canis* [45].

## 4. Agreement of CLSI and Agar-Based Diffusion Methods

The agreement analysis between the results (i.e., MIC value of specific drugs) obtained with CLSI standard reference procedures and agar-based diffusion methods (i.e., DD and E-Test) are useful to determine if agar-based diffusion methods could be an alternative of CLSI reference procedures for use in clinical laboratories. High levels of agreement were described for yeasts and some important filamentous fungi [65] but for dermatophytes, this needs to be better defined. Only very few studies are published on this issue and the results show that the level of agreement between these methods are drug dependent [66]. In particular, low levels of agreement were observed using KTZ, FLZ, and GRI [41,64]. It is well known that MICs generated using agar-based techniques tended to be much higher than those produced by broth assays [48,59]. These data suggest caution in interpreting the MICs of the above drugs, which may falsely indicate resistance in the agar based method and falsely suggest susceptibility in broth microdilution. The best of these methods that is more predictive of successful outcome need to be better addressed by evaluating the clinical efficacy of the drugs and the susceptibility profile of the fungus causing the infection. However, the development of CLSI standardised reference method with which these agar-based methods can be compared is warranted.

## 5. Antifungal Resistance

Antifungal resistance can be defined as microbiologic or clinical resistance [67]. Microbiological resistance occurs when growth of the infecting organism is inhibited by a drug concentration higher than the range seen for wild-type strains, whereas clinical resistance when the infecting organism is inhibited by a concentration of an antifungal agent that is associated with a high likelihood of therapeutic failure. 

Although, resistance mechanisms are considered a threat for many fungal species (i.e., *Aspergillus and Candida* spp.), the antifungal resistance (i.e., microbiologic and clinical resistance) in dermatophytes is seldom reported and verified only in *T. rubrum* [68,69], *T mentagrophytes* [70], and more recently, in *M. canis* [32].

*M. canis* infection had always been simple to treat with antifungal agents. However, there has been a sudden increase in the number of patients with recalcitrant infection [71] and clinical resistance is usually suspected when a clinical case presents with persistent infection or relapses within 4 weeks of an adequate dose regimen of an antifungal drug [13]. This is based on the skin pharmacokinetics (PK) of the major drugs used in dermatophytosis since usually they permeate into the stratum corneum for 3 to 4 weeks after discontinuation of therapy [72,73]. However, different other causes might be associated with relapsing and they are related to drug interactions, poor patient compliance, overwhelming infection, difficult-to-reach site of infection, wrong administration of drugs, underlying disorder interfering with the immune system, and lack of environmental control [24,74].

Furthermore, the dermatophytes disorders are also considered as recalcitrant infection with tinea capitis caused by *Microsporum canis* as one of the most important and with an increasing incidence during the last decade [13]. Recalcitrant fungal infections are those infections that are difficult or impossible to eradicate after adequately treated with appropriate oral or parenteral antifungal agents [13]. Relative or absolute microbial and clinical resistance, failure of the patient to comply with the prescribed treatment regimen, drug degradation in the liver, drug–drug interactions, reabsorption or washout of the drug from the stratum corneum, and failure of the drug to reach the stratum corneum are all causes of recalcitrance [75]. Regardless of microbiological resistance of *M. canis*, no interpretative breakpoints are available, thus resistance phenomena should only be suspected when MIC values of the tested drugs are in the range established for *Candida* spp. and *Aspergillus* spp. resistant strains. In particular, for FLZ, the strains with MICs greater than or equal to 64 µg/mL should be considered resistant [76,77]. On this respect, for *M. canis*, high MIC values for FLZ were reported by using broth microdilution or agar-based methods, thus indicating that FLZ is not the best choice for curing *M. canis* induced disorders since resistance phenomena for FLZ in *M canis* might be suspected [48,50,51,56,64]. Similarly, high MIC values and low inhibition zone diameters were reported for GRI by using both broth microdilution and agar based methods [45,53]. In particular, MIC values higher than 3 μg/mL and a diameter of inhibition zone lower than 26 mm (25 µg/disk) were previously registered in *M. canis* strains coming from humans [45]. Since these values are considered a concentration limit for the effectiveness of therapy for *T*. *rubrum* [78], GRI should not be the best option for treatment of *M*. *canis* infection both in humans and animals. On the contrary, it seems that TER and ITZ are the more active drugs when compared with GRI and other azoles on the basis of MIC values obtained with different protocols [50,51,56,64] and should be the best treatment option for *M. canis* infection. 

## 6. Conclusions

Available data for both the current conventional therapy options of *M. canis* infections and the in vitro antifungal profiles of *M. canis* obtained using agar-based and microbroth dilution methods have been herein reported to help in future establishment of methods and clinical breakpoints for *M. canis*. Recalcitrant and recurrence phenomena of *M. canis* infections were well-documented but they were never related with the microbiological resistance of the causative agent, thus making not possible to establish clinical breakpoints. In addition, it becomes clear that susceptibility tests for dermatophytes in clinical practice should be standardised to compare results from various reports. However, some of the testing options, using modified CLSI microdilution methods, such as PDA medium, incubation temperature of 28 ± 2 °C for 7 to 14 days [59] appear suitable and worth adopting in a future reference standard dedicated to this fungus. An inoculum of 10^4^ CFU/mL and incubation for 7 days at 28 °C should be the best option for reading of the MIC values in CLSI procedures. Most stringent MIC determination criterion (MIC-0 = 100%) should be the best criteria because of the reproducibility of data among intra- and interlaboratories results [37,59]. When a standard procedure is obtained, the results of agar diffusion methods should be better compared. Specifically for DD, the efficacy of MH or RPMI agar in testing the efficacy of the drugs should be better clarified and the usefulness of RPMI agar or other media in E-test should be also ascertained. At the moment, until a reference method for testing the in vitro susceptibility of dermatophytes is standardised, we could suggest which drug is active on the basis of its MIC value obtained with a particular method. Finally, since the low activities of FLZ and GRI is a constant finding regardless of the methods employed to evaluate the MIC results, it should be noted that FLZ and GRI might not be an optimal choice when a long term therapy is expected to be used during the treatment of this clinical infection in humans and animals.

## Figures and Tables

**Table 1:** In vivo prospective studies on the topical and systemic treatment of animal dermatophytosis reporting clinical and/or mycological outcome.

References	Agent Tested	Protocol	Length of Treatment	Animals	RCT	Blinded	Outcome: Improvement in Clinical Signs and Mycology
[25]	Enilconazole + Griseofulvin (group 1) vs. Enilconazole + Luferunol (group 2)	0.2% enilconazole weekly, topically; Griseofulvin 25 mg/kg/BID, PO; Lufenuron 60 mg/kg PO two administration one month apart.	1 month	100 cats (group 1 36 cats; group 2 64 cats)	No	No	Failure
[26]	Griseofulvin vs. Miconazole/Chlorhexidin + Griseofulvin	Griseofulvin: 50 mg/kg/SID PO; 2% Miconazole/2% Chlorhexidine, topically	2 ½ months	14 cats (7 griseofulvin; 7 griseofulvin + Miconazole/Chlorhexidine	No	No	Complete 14/14 with both (lesion in the group receiving topical therapy decreased more quickly than in the group receiving systemic therapy alone)
[27]	Griseofulvin + Miconazole/Chlorhexidine vs. Griseofulvin alone	Griseofulvin 50 mg/kg/SID, PO; 2% Miconazole/2% Chlorhexidine, topically	3 months	21 cats	No	No	Complete 21/21 (benefit from the addition of twice-weekly chlorhexidine-miconazole shampooing to systemic griseofulvin therapy alone)
[28]	Griseofulvin vs. Itraconazole vs. Control	Griseofulvin: 50 mg/kg/SID, PO (group 1); Itraconazole: 10 mg/kg/SID, PO, (group 2); Control (group 3)	3 months	15 cats (5 group 1; 5 group 2; 5 group 3)	Yes	No	Complete 10/15 (itraconazole-treated group was the first to achieve a cure, followed by the griseofulvin-treated group)
[29]	Terbinafine	30 mg/kg/SID, PO	14 days	12 cats	No	No	Complete 11/12
[30]	Terbinafine	8.25 mg/kg/SID, PO	21 days	9 cats	No	No	Complete 9/9
[31]	Terbinafine	10–20 mg/kg/SID (group 1); 30–40 mg/kg/SID (group 2) PO	4 months	18 cats (9 group 1; 9 group 2)	No	No	Complete 18/18

**Table 2:** CLSI procedures for antifungal susceptibility in *Microsporum canis*. Range of MIC values (µg/mL) of azoles, griseofulvin, and terbinafine were also reported.

*M. canis* Strains	Pre-Incubation (Days-°C)/Media	Type of Inoculum/Inoculum Size	Medium	% of Inhibition	MIC Range Value							Incubation (Days/°C)	References
					FLU	IT	KTZ	TER	GRI	POS	VOR		
5	4–5 days-30 °C/PDA	Hypha + conidia/0.5–4 × 10^4^	RPMI	50	0.125 ≥ 64	0.001–0.125		0.001 ≥ 0.5	0.125–64	0.015–0.125	0.001–0.25	35 °C/4 days	[37]
11	4–7 days-30 °C/SDB	Hypha + conidia	SAB	50	nd	nd	nd	nd	<0.25–16	nd	nd	37 °C/14 days	[38]
7	7–10 days/30 °C/PDA	Hypha + conidia/1–2 × 10^4^	RPMI	50	0.5–2	0.03–1	nd	0.002–0.125	0.06–2	0.03–0.5	nd	30 °C/5 days	[39]
19	7 days/28 °C/PDA	Hypha + conidia/1–2 × 10^4^	RPMI	80	2–32	0.03–4	0.03–4	0.03–1	0.06–8	nd	nd	28 °C/5 days	[40]
20	14 days/27 °C/PDA	Hypha + conidia/0.4–5 × 10^4^	RPMI	80	-	0.06–4	0.125–16	0.03–16	nd	nd	nd	27 °C/3 days	[41]
16	7–10 days/28 °C/	Hypha + conidia	RPMI	50	0.625–256	0.0009–0.5	0.0625–4	0.03–8	0.02–128	nd	0.02-8	28 °C/7 days	[14]
94	7 days/30 °C/PDA	Conidia//0.5–4 × 10^4^	RPMI	nd	nd	nd	nd	0.004–0.25	nd	nd	nd	35 °C/4 days	[42]
9	SDA	Conidia	RPMI	80	0.06–128	nd	nd	64–256	nd	nd	nd	35 °C/7 days	[43]
1	14 days/30 °C/PDA	Conidia//0.5–5 × 10^4^	RPMI	100	0.01–64	nd	nd	nd	nd	nd	-nd	30 °C/4 days	[44]
7	7 days/28 °C/SDA	Conidia/10^3^–10^4^	RPMI	50	0.03–64	0.03–16	0.03–16	0.03–16	0.03–16	nd	nd	28 °C/5 days	[45]
2	7 days/30 °C/PDA	nr	RPMI	90	2–4	0.125	nd	nd	2	nd	nd	35 °C/4–5 days	[46]

SDB: Sabouraud Dextrose Broth; SDA: Sabouraud Dextrose Agar; Agar PDA: Potato Dextrose Agar; nd: not done; nr: not reported.

**Table 3:** E-test procedures for antifungal susceptibility in *Microsporum canis*. Range of MIC values (µg/mL) of azoles, grisefulvin, and terbinafine were also reported.

*M. canis* Strains	Pre-Incubation (Days-°C)/Media	Type of Inoculum/Inoculum Size	Medium	MIC Range Value							Incubation (Days/°C)	References
				FLU	IT	KTZ	TBN	GRI	POS	VOR		
	7 days/28 °C/PDA	Hypha + conidia/10^6^ cells/mL	SDA	>256	>32	>32	nd	nd	nd	nd	25 °C/3–5 days	[47]
6	10–15days–28 °C/PDA	Hypha + conidia/10^5^–10^6^ cfu/mL	RPMI agar	>256	0.25–1	0.125–1	nd	nd	nd	nd	28 °C/3 days	[48]
20	14 days–27 °C/PDA	Hypha + conidia/10^5^–10^6^ cfu/mL	RPMI agar	nd	0.064–1	0.19–0.75	nd	nd	nd	nd	27 °C/4 days	[41]
5	15 days–28 °C/PDA	Hypha + conidia/10^5^–10^6^ cfu/mL	RPMI agar	2–8	1–32	32	nd	nd	nd	nd	28 °C/3–4 days	[49]

SDA: Sabouraud Dextrose Agar; nd: not done.

**Table 4:** Disk diffusion procedures for antifungal susceptibility in *Microsporum canis*. Inhibition Zone Diameter (mm) for azoles, Griseofulvin and terbinafine were also reported.

*M. canis* Strains	Pre-Incubation Days/°C/Media	Type of Inoculum/Inoculum Size	Medium Agar	MIC range values (mm)							Incubation (Days/°C)	References
				FLU (µg/disk)	ITZ (µg/disk)	KTZ (µg/disk)	TER (µg/disk)	GRI (µg/disk)	POS (µg/disk)	VOR (µg/disk)		
34	7–14/28 °C/PDA	Hypha + conidia/10^4^–10^6^ cells/mL	PDA	<14–≥22 (25)	0 ≥ 16 (10)	nd	nd	nd	nd	≥14 (1)	28 °C/2–7	[50]
10	14/28 °C/PDA	Hypha + conidia	SAB	nd	22–48	nd	83–89	nd	nd	nd	28 °C/7	[51]
5	7–14/27 °C/PDA	Hypha + conidia/10^4^ cells/mL	RPMI	nd	20–26 (2)	21–27 (0.005)	nd	nd	nd	25–33 (0.005)	28 °C/5	[45]
7	7–10/30 °C/PDA	Hypha + conidia/10^6^ cells/mL	Dermasel	0–0 (25)	14–20 (10)	nd	56–72 (1)	40–50 (10)	22–32 (5)	nd	30 °C/4–7	[42]
8	4–15/30 °C/PDA	Hypha + conidia/10^6^ cells/mL	MH	0–0 (25)	25–40 (8)	15–40 (15)	36–67 (1)	35–68 (10)	nd	45–68 (1)	37 °C/14	[52]
19	14/30 °C/PDA	Hypha + conidia	RPMI	nd	0–38 (10)	0–62 (15)	nd	0–82 (25)	nd	nd	30 °C/14	[53]
7	7–10/28 °C/SDA	Conidia/×10^4^ cells/mL	MH-glucose-Methylene blue	nd	23.0 ± 0.25 (10)	22.0 ± 0.25 (10)	16.0 ± 0.12 (30)	16.0 ± 0.2 (25)	nd	nd	28 °C/7	[45]
58	28/25 °C/Dermasel	Hypha + conidia	MH	<14–≥22 (25)	nd	nd	nd	0–>10 (10)	nd	nd	25 °C/5–10	[54]

nd: not done.
